# Lung Adenocarcinoma Presenting With Elevated Serum Carbohydrate Antigen 19-9 Levels: A Case Report

**DOI:** 10.7759/cureus.77786

**Published:** 2025-01-21

**Authors:** Mónica Lopes, Vera Figueiredo, Ana Mendes, Marta Amaral, José Delgado Alves

**Affiliations:** 1 Department of Internal Medicine IV, Hospital Professor Doutor Fernando Fonseca, Amadora, PRT; 2 Department of Pulmonology, Hospital Professor Doutor Fernando Fonseca, Amadora, PRT; 3 Department of Oncology, Hospital Professor Doutor Fernando Fonseca, Amadora, PRT

**Keywords:** cancer antigen 19-9, elevated ca 19-9, lung cancer with gastrointestinal features, metastatic non-small cell lung cancer, tumor biomarkers

## Abstract

Carbohydrate antigen 19-9 (CA 19-9) is a tumor marker usually used for the diagnosis and follow-up of pancreatic, gastric, and hepatobiliary malignancies. However, it has a low specificity and can be elevated in a wide array of other conditions. CA 19-9 elevation in lung tumors seems to be associated with the worst prognosis, but its role in this condition is not fully established yet. We present the case of a 78-year-old woman with mild consumptive symptoms, consisting of unselective anorexia and weight loss, and isolated high levels of CA 19-9. Hepatobiliary and gastrointestinal lesions were excluded, and a suspicious lesion was found in the right lung. A biopsy, which was only possible several months after presentation due to the location and small dimensions of the lesion, confirmed the diagnosis of lung adenocarcinoma. After an initial indolent course, the patient evolved with disease progression and is currently under treatment with palliative chemotherapy. This case illustrates the thorough investigation performed to clarify the elevation of a tumor biomarker measured in a pauci-symptomatic patient, which led to the identification of a rare and unsuspected etiology of high levels of CA 19-9.

## Introduction

Carbohydrate antigen 19-9 (CA 19-9) is a sensitive tumor marker for pancreatic, gastric, and hepatobiliary malignancies [1-3]. Levels of CA 19-9 exceeding 1000 U/mL are highly suggestive of malignancy in the adequate clinical setting and indicative of a poor prognosis [1-3]. CA 19-9 may also be overexpressed in other gastrointestinal, urological, pulmonary, or gynecological cancers, as well as in benign diseases, especially involving the hepatobiliary and pulmonary tissues [4-8].

Lung tumors are usually associated with the elevation of other tumor biomarkers, such as cytokeratin 19 fragment (CYFRA 21-1) and neuron-specific enolase (NSE) [4]. It can also be associated with elevated levels of CA 19-9, but its use for diagnostic purposes is not recommended, especially in asymptomatic patients [4,9]. As a prognostic marker in lung cancers, its elevation is apparently associated with advanced disease stages, shorter survival times, and a higher risk of disease progression, but further investigation is needed to establish its routine assessment [9,10].

Screening of tumor biomarkers such as CA 19-9 is becoming more frequent as general health check-ups become widely available, but its use in asymptomatic or only mildly symptomatic patients (particularly with unspecific symptoms) is not recommended [8]. When ordered, if elevated levels of CA 19-9 are found, there are no guidelines regarding subsequent workup [5-8].

We present the case of a 78-year-old woman with mild consumptive symptoms and elevated CA 19-9 levels. Exhaustive complementary workup led to the unexpected diagnosis of a lung adenocarcinoma.

## Case presentation

A 78-year-old woman was referred to our hospital with a one-month history of anorexia, malaise, and weight loss of 5 kg, and a level of CA 19-9 of 12,603 U/mL (normal range: <39 U/mL). She had a previous history of valvular and ischemic cardiomyopathy and had been submitted to aortic valve replacement two years before. She had also type 2 diabetes mellitus and arterial hypertension, both adequately controlled. She denied smoking. Considering this, she was admitted for further workup.

Her physical examination was unremarkable. Her laboratory results showed only slightly elevated aspartate aminotransferase (75 U/L, normal range: <32 U/L), alanine aminotransferase (62 U/L, normal range: <33 U/L), alkaline phosphatase (150 U/L, normal range: 35-105 U/L), and gamma-glutamyl transpeptidase (200 U/L, normal range: <40 U/L), without hyperbilirubinemia (Table 1).

**Table 1:** Patient laboratory evaluation at admission PT: prothrombin time; INR: international normalized ratio; aPTT: activated partial thromboplastin time; CA 19-9: carbohydrate antigen 19-9

Parameter	Patient values	Reference range
Hemoglobin (g/L)	13.3	12.0-15.0
Leucocyte (x10^9^/L)	5.6	4.0-10.0
Platelets (x10^9^/L)	177	150-410
PT (seconds)	11.3	9.7-11.8
INR	1.1	<1.2
aPTT (seconds)	24.9	20.6-29.5
Sodium (mmol/L)	141	136-145
Potassium (mmol/L)	3.77	3.5-5.1
Chloride (mmol/L)	101.1	98-107
Calcium (mg/dL)	9.3	8.8-10.2
Phosphate (mg/dL)	3.5	2.5-4.5
Magnesium (mg/dL)	1.8	1.6-2.6
Aspartate aminotransferase (U/L)	86	<32
Alanine aminotransferase (U/L)	50	<33
Alkaline phosphatase (U/L)	175	35-105
Gamma-glutamyl transpeptidase (U/L)	286	<40
Total bilirubin (mg/dL)	0.6	<1.2
Lactate dehydrogenase (U/L)	254	135-214
Creatinine (mg/dL)	0.87	0.7-1.2
Urea (mg/dL)	46	<50
Albumin (g/dL)	4.1	3.97-4.94
Creatine kinase (U/L)	63	39-308
Reactive C protein (mg/dL)	1.01	<0.5
CA 19-9 (U/mL)	12,603	<39

**Figure 1:**
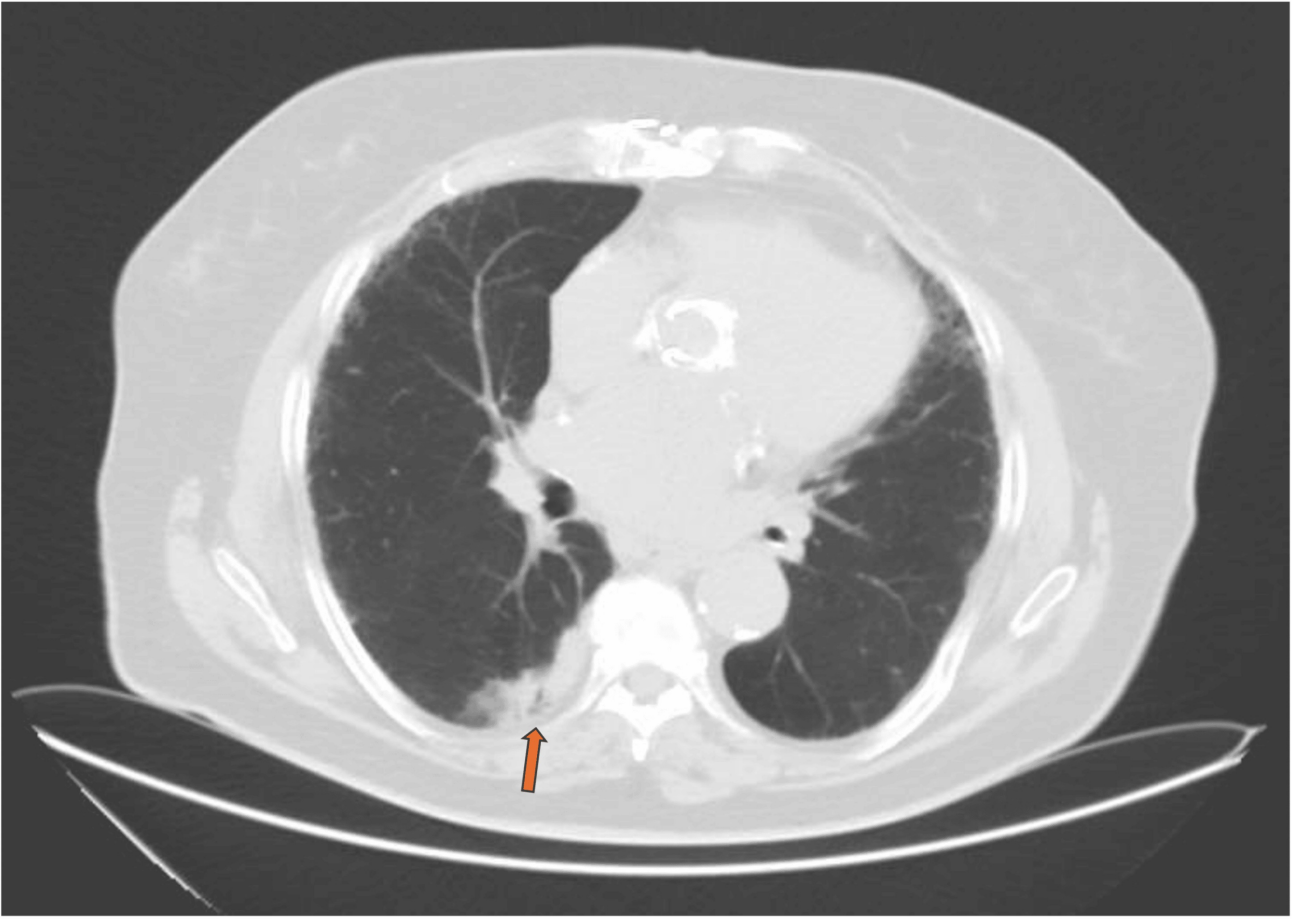
Chest CT scan showing a heterogeneous condensation in the posterior aspect of the lower right lobe (orange arrow)

A thoraco-abdomino-pelvic computed tomography (CT) scan only showed diffuse pulmonary emphysema and a heterogenous condensation in the lower right lobe, suggestive of an infectious process sequela (Figure 1).

Common gastrointestinal neoplasms were excluded with a digestive endoscopy and a colonoscopy. A magnetic resonance cholangiopancreatography (MRCP) excluded lesions in the biliary tract. Pelvic examination and endocavitary ultrasound were normal. Urinary cytologic examination showed some atypical urothelial cells, but direct cystoscopy did not reveal pathologic findings, and a urologic CT scan excluded alterations in the urinary system.

During hospitalization, the levels of CA 19-9 decreased (1984 U/mL was the minimum value), and she regained her appetite and the weight she had lost. Given this, the hypothesis of malignancy was considered unlikely, and she was discharged.

Despite this benign evolution, we requested a positron emission tomography with computed tomography (18F-FDG PET/CT) that revealed densification in the inferior right pulmonary lobe and positive adenopathic peribronchial infiltration from that area up to the mediastinum (stations 2R, 4R, 7, 10, and 11), with standardized uptake values (SUV) ranging from 5 to 14. Endobronchial ultrasound (EBUS) allowed a biopsy of three adenopathies that showed suspicious cells but with insufficient material for a complete diagnosis. A transthoracic needle biopsy (TNB) of the lung lesion was attempted, but the sample showed unspecific changes.

She was evaluated by the cardiothoracic surgery team for mediastinoscopy approximately six months after the first TNB. At this time, a new thoracic CT scan showed a slight increase in the pulmonary lesion, making it accessible. A second TNB was performed, and histology was compatible with adenocarcinoma with CK7 and CDX-2 expression but TTF1, CK20, GATA3, ER, and p40 were negative. The primary suspicion was a lung neoplasm; thus, the required anatomopathological and molecular studies were conducted, revealing a PD-L1 TPS (Ventana 22C3) expression of <1% and the amplification of the HER2 gene (22.56x).

To complete staging, biopsies of the mediastinum adenopathies were performed once more with EBUS, this time confirming the presence of malignant cells. Given her high surgical risk and the confirmed multilevel disease, surgical resection of the mass was deemed unfeasible. The patient progressed with anorexia and right pleuritic thoracalgia. A thoraco-abdomino-pelvic CT scan performed at this time (and approximately 18 months after initial admission) showed metastasis in both lungs and the right adrenal gland. Considering her age and comorbidities, which conferred her an Eastern Cooperative Oncology Group (ECOG) performance score of 2, monotherapy with gemcitabine was proposed. At the time of this report, she completed the first cycle of gemcitabine without immediate complications.

## Discussion

Serum tumor biomarkers are important to diagnose cancer in high-risk patients and allow evaluation of treatment response and early detection of cancer recurrences [1-3]. The increasing accessibility of health checkups is leading to an increase in tumor biomarker screening in healthy individuals, although these are not validated for this purpose [11,12].

Serum CA 19-9 is commonly elevated in patients with pancreatic cancer and other gastrointestinal malignancies [1-3]. This biomarker may also rise in other malignant and benign conditions, such as liver cirrhosis, pancreatitis, genitourinary tumors, chronic kidney failure, rheumatic and thyroid diseases, diabetes mellitus, and benign lung conditions [4-8].

Levels of CA 19-9 can be useful in detecting cancer in symptomatic patients (with significant weight loss, jaundice, or abdominal pain) or when imaging studies show pancreatic or hepatobiliary masses [5]. In asymptomatic individuals, CA 19-9 screening is not advisable due to its very low positive predictive value (less than 2%), making it ineffective and costly as a diagnostic marker in this group [8,11,13]. In our patient, CA 19-9 was ordered to investigate vague symptoms that rapidly subsided. This was not appropriate considering the existing evidence [5,6,8,11-13].

There are no guidelines regarding subsequent workup in asymptomatic patients with elevated CA 19-9 [5-8]. After ruling out pancreatic or hepatobiliary tumors, further laboratory and imaging studies, including liver and thyroid function, blood sugar, a chest CT scan, and a revision of the abdominopelvic CT scan to exclude gynecologic disease and endoscopic studies, are suggested [5,8]. If no potential causes are identified, a close follow-up of the serum levels of CA 19-9 is recommended [5-8]. In this case, the very high levels of CA 19-9 led us to do a thorough study to exclude malignancy, especially considering the patient’s age and her consumptive symptoms.

The magnitude of the elevation of CA 19-9 levels can reflect the nature of the underlying condition. Usually, benign diseases are associated with lower levels of serum CA 19-9 (<500 U/mL) that remain stable or decrease during follow-up [5-7]. Furthermore, when the suspected benign condition is controlled, the levels of CA 19-9 tend to normalize [5-8]. Conversely, in malignant diseases, these levels can significantly rise after a short follow-up period [5-7]. Interestingly, our patient showed a significant decrease in the value of CA 19-9, alongside symptomatic improvement, during her admission. Levels of CA 19-9 rose again when the disease progressed.

Lung cancer is a prevalent solid tumor frequently associated with a poor prognosis, mainly due to its late-stage detection [14]. Literature on the role of serum tumor biomarkers for lung cancer diagnosis is scarce, and, so far, none is recommended for this purpose [13,15]. Carcinoembryonic antigen (CEA), CYFRA 21-1, and NSE seem to be useful in the presence of suspicious lung findings or for early detection of lung cancer in patients at risk, in association with low-dose chest CT scans [4,15,16]. The usefulness of other biomarkers in lung cancer diagnosis, such as the squamous carcinoma cell antigen (SCCA), the cancer antigen 125 (CA 125), and CA 19-9, is not clear, and different authors present contradicting results [4,9].

In the case presented, gastrointestinal and genitourinary malignancies, which are more frequently associated with high levels of CA19-9, were excluded first. In the absence of abnormal findings in the extensive workup performed, we considered that measuring other tumor biomarkers would not be useful. The lung changes found in the first CT scan were not suspicious, and these findings were only pursued when the PET scan showed abnormal metabolic activity in this territory. In reported studies with large groups of patients with elevated levels of CA 19-9, lung tumors were diagnosed in less than 0.6% of patients, which illustrates the rarity of this etiology in this setting [5,7,8].

Due to the small dimensions and inaccessibility of the lung lesion, a viable biopsy was only possible more than six months after the first presentation, which coincided with the patient’s clinical deterioration. Reported delays between finding elevated levels of CA 19-9 and diagnosing a malignancy can reach two years after the first altered value [5]. Hence, it is not clear that high levels of CA 19-9 lead to an early diagnosis of lung tumors. 

Some studies suggest that elevated levels of CA 19-9 indicate a shorter overall survival and higher risk of disease recurrence in lung cancer and thus propose its use as a prognostic marker, particularly in combination with other biomarkers such as CYFRA 21-1 [9,10]. The absence of TTF-1 staining in the lung specimen, which is not infrequent in lung adenocarcinoma, is also a poor prognosis marker [17]. In our patient, who presented with very mild symptoms, there was a prolonged initial period during which no relevant imaging or clinical progression occurred. However, after this, she evolved with a rapidly progressive and metastatic disease, which is in line with the altered biochemical markers found.

## Conclusions

CA 19-9 can be a valuable tumor biomarker, but its specificity is very limited, and it should not be measured in asymptomatic or pauci-symptomatic patients. Interpretation of elevated levels of CA 19-9 and further workup should be individualized. The value of CA 19-9 in lung cancers is not fully established. It does not seem useful for diagnosis, but it may be valuable to predict long-term prognosis.

Our case is particularly interesting because the diagnosis of malignancy in asymptomatic patients with elevated levels of CA 19-9 is infrequent, and, within this group, lung tumors are rare. Furthermore, the initial indolent course of this patient and the decreasing trend of the CA 19-9 levels were both atypical, considering the nature of the underlying tumor. The aggressive evolution afterward seems to be more in line with the very high levels of CA 19-9 seen initially.

## References

[1] Zhu JW, Gong LZ, Wang QW (2024). Serum tumor markers (carcinoembryonic antigen, carbohydrate antigen 19-9, carbohydrate antigen 72-4, carbohydrate antigen 24-2, ferritin) and gastric cancer prognosis correlation. World J Gastrointest Surg.

[2] Zhang Y, Yang J, Li H, Wu Y, Zhang H, Chen W (2015). Tumor markers CA19-9, CA242 and CEA in the diagnosis of pancreatic cancer: a meta-analysis. Int J Clin Exp Med.

[3] Kim NH, Lee MY, Park JH, Park DI, Sohn CI, Choi K, Jung YS (2017). Serum CEA and CA 19-9 levels are associated with the presence and severity of colorectal neoplasia. Yonsei Med J.

[4] Sun A (2023). Clinical role of serum tumor markers SCC, NSE, CA 125, CA 19-9, and CYFRA 21-1 in patients with lung cancer. Lab Med.

[5] Kim S, Park BK, Seo JH (2020). Carbohydrate antigen 19-9 elevation without evidence of malignant or pancreatobiliary diseases. Sci Rep.

[6] Kim BJ, Lee KT, Moon TG, Kang P, Lee JK, Kim JJ, Rhee JC (2009). How do we interpret an elevated carbohydrate antigen 19-9 level in asymptomatic subjects?. Dig Liver Dis.

[7] Tong Y, Song Z, Zhu W (2013). Study of an elevated carbohydrate antigen 19-9 concentration in a large health check-up cohort in China. Clin Chem Lab Med.

[8] Lee SP, Sung IK, Kim JH, Lee SY, Park HS, Shim CS (2019). Usefulness of carbohydrate antigen 19-9 test in healthy people and necessity of medical follow-up in individuals with elevated carbohydrate antigen 19-9 level. Korean J Fam Med.

[9] Isaksson S, Jönsson P, Monsef N (2017). CA 19-9 and CA 125 as potential predictors of disease recurrence in resectable lung adenocarcinoma. PLoS One.

[10] Sato Y, Fujimoto D, Uehara K (2016). The prognostic value of serum CA 19-9 for patients with advanced lung adenocarcinoma. BMC Cancer.

[11] Locker GY, Hamilton S, Harris J (2006). ASCO 2006 update of recommendations for the use of tumor markers in gastrointestinal cancer. J Clin Oncol.

[12] Wang HY, Hsieh CH, Wen CN, Wen YH, Chen CH, Lu JJ (2016). Cancers screening in an asymptomatic population by using multiple tumour markers. PLoS One.

[13] Kim JE, Lee KT, Lee JK, Paik SW, Rhee JC, Choi KW (2004). Clinical usefulness of carbohydrate antigen 19-9 as a screening test for pancreatic cancer in an asymptomatic population. J Gastroenterol Hepatol.

[14] Bray F, Laversanne M, Sung H, Ferlay J, Siegel RL, Soerjomataram I, Jemal A (2024). Global cancer statistics 2022: GLOBOCAN estimates of incidence and mortality worldwide for 36 cancers in 185 countries. CA Cancer J Clin.

[15] Stieber P, Hatz R, Holdenrieder S (2006). National Academy of Clinical Biochemistry guidelines for the use of tumor markers in lung cancer. Tumour Biol.

[16] Triphuridet N, Vidhyarkorn S, Worakitsitisatorn A (2018). Screening values of carcinoembryonic antigen and cytokeratin 19 fragment for lung cancer in combination with low-dose computed tomography in high-risk populations: initial and 2-year screening outcomes. Lung Cancer.

[17] Iso H, Hisakane K, Mikami E (2023). Thyroid transcription factor-1 (TTF-1) expression and the efficacy of combination therapy with immune checkpoint inhibitors and cytotoxic chemotherapy in non-squamous non-small cell lung cancer. Transl Lung Cancer Res.

